# Supporting health education policies: translation, cross-cultural adaptation and validation of a health literacy instrument, in French

**DOI:** 10.3389/fpubh.2023.1326771

**Published:** 2023-12-21

**Authors:** Céline Clément, Virginie-Eve Lvovschi, Elise Verot, Benjamin du Sartz de Vigneulles, Adeline Darlington-Bernard, Denis Bourgeois, Michel Lamure, Flavia Vitiello, Claude Dussart, Florence Carrouel

**Affiliations:** ^1^Laboratory “Health, Systemic, Process” Laboratory (P2S), UR4129, University Claude Bernard Lyon 1, University of Lyon, Lyon, France; ^2^Laboratory “Interpsy”, UR 4432, University of Lorraine, Nancy, France; ^3^Laboratory “Research on Healthcare Performance” (RESHAPE), INSERM U1290, University Claude Bernard Lyon 1, Lyon, France; ^4^Hospices Civils of Lyon, Lyon, France; ^5^PRESAGE Institute, University Jean Monnet, Saint-Etienne, France; ^6^CIC 1408 Inserm, CHU of Saint-Etienne, Saint-Etienne, France; ^7^Department of Clinical Sciences and Stomatology (DISCO), University Politecnic of Marche, Ancona, Italy

**Keywords:** literacy, public health, oral disease, questionnaire, validation, France

## Abstract

**Background:**

Oral health is a fundamental human right and is inseparable and indivisible from overall health and well-being. Oral Health Literacy (OHL) has been proved to be fundamental to promoting oral health and reducing oral health inequalities. To our knowledge, no OHL instrument to evaluate OHL level is currently validated in French language despite the fact it is the fifth most widely spoken languages on the planet. The Oral health literacy Instrument (OHLI) appears to be the most interesting OHL instrument to adapt into French because it is already available in English, Spanish, Russian, Malaysian, and it contains both reading comprehension and numeracy sections. Its psychometric properties have been rated as adequate.

**Objective:**

The aim of this study was to translate and adapt cross-culturally the OHLI into French, to evaluate its psychometric properties and to compare its results to oral health knowledge.

**Method:**

This study followed and applied well-established processes of translation, cross-cultural adaptation and validation, based on the recommendations of the World Health Organization guidelines and on the Consensus-Based Standards for the Selection of Health Measurement Instruments (COSMIN) study design checklist for patient-reported outcomes. Two psychometric assessments were planned, the comparison of OHLI-F scores according to education level and frequency of dental visits, and the test–retest reliability of the OHLI-F.

**Results:**

A total of 284 participants answered the OHLI-F. The OHLI-F scores were significantly different between participants with different levels of education and frequency of dental visits (*p* < 0.001). Participants with an education level lower than the baccalaureate, and those who never visit the dentist or only in case of pain, had significantly lower OHLI-F scores. Internal consistency was excellent (Cronbach’s alpha = 0.881–0.914). Test–retest reliability was very high (intraclass correlation = 0.985 to 0.996).

**Conclusion:**

The OHLI-F has demonstrated adequate psychometric properties and can therefore be used to measure oral health literacy in French-speaking populations.

## 1 Introduction

Oral health is a fundamental human right and is inseparable and indivisible from overall health and well-being (1). However, public and professional discourses often associate oral health with the presence or absence of oral disease rather than adopting a holistic, person-centered focus, and disease treatment becomes the primary aim (2). As a consequence, the global burden of oral diseases has amounted to an annual expenditure of about US$ 387 billion in direct costs and another US$ 323 billion in indirect costs (3). In many communities, particularly in socially marginalized groups, and older people, oral diseases remain largely untreated because of difficult access to dental care and treatment costs exceeding available resources (4). In Europe, oral diseases persist with high prevalence, reflecting social and economic inequalities and inadequate funding for prevention and treatment (5).

As most non-communicable diseases, oral diseases are largely preventable. Higher standards in oral hygiene can be achieved through education, teaching, motivation, oral hygiene instructions, and improving people’s skills and attitudes toward their oral health (6). Indeed, patients play an active role on their own health and responsibility, and compliance is crucial (7).

Health Literacy (HL) is defined as “the knowledge, motivation and competences to access, understand, appraise and apply health information in order to make judgments and take decisions in everyday life concerning health care, disease prevention and health promotion to maintain or improve quality of life throughout the course of life” (8). Across countries, specific populations have a proportion of people with more limited HL than the general population, suggesting the existence of specific vulnerable groups (9). Like HL, Oral Health Literacy (OHL) has also proven to be fundamental to promoting oral health and reducing oral health inequalities (10), as well as contributing to overall health and well-being (11–13). Despite the fact data about the status of OHL in Europe remains scarce, it has been demonstrated that financial conditions, followed by social status, education and age appears to be a strong predictor of low OHL (11).

To evaluate OHL, several instruments are available such as the Rapid Estimate of Adult Literacy in Dentistry (REALD-30) (14), the Oral Health Literacy Instrument (OHLI) (15), the Test of Functional Health Literacy in Dentistry (TOFHLiD) (16), and the Comprehensive Measure of Oral Health Knowledge (CMOHK) (12). To date, most of these available OHL instruments are principally based on the assessment of functional literacy, through timed tests which evaluate the recognition or the understanding of medical terms. Their objectives are often restricted, some are very long and others include items that are not relevant to all societies (17).

To our knowledge, no validated OHL instrument is available in French language. French is the fifth most widely spoken languages on the planet after English, Chinese, Hindi and Spanish (18). It is the official language of four European countries (France, Belgium, Switzerland, and Luxembourg) and of 25 independent nations outside Europe (18). As for any measurement tool, measurement invariance is a required property to guarantee accurate group comparisons and is thus essential for questionnaire validation. The OHLI appears to be the most interesting to adapt into French. A recent systematic review highlighted that the OHL instruments originally developed for English speakers lack cultural and linguistic sensitivity when applied to non-English-speaking populations (19) and some items are not relevant to all population groups (20). Thus, “cross-cultural adaptation” (language (translation) and cultural adaptation) of the instrument is necessary for use it in a new country (21, 22). This makes it possible to preserve equivalence with the original instrument and help to check whether the adapted version of the instrument retains its psychometric properties (23, 24).

Indeed, OHLI is already available in English (25) Spanish (26), Russian (27), Malaysian (28), and contains both reading comprehension and numeracy sections to measure a person’s ability to perform OHL tasks, and the psychometric properties of these adapted versions have been rated as adequate (15, 28).

Thus, the objective of this study was to translate and adapt cross-culturally the OHLI into French, to evaluate its psychometric properties and to compare its results to oral health knowledge in an adult population.

## 2 Materials and methods

### 2.1 Study design

This study followed and applied a well-established process of translation, cross-cultural adaptation and validation based on the recommendations of the World Health Organization (29) and the guidelines developed by Beaton et al. (21). In addition, to select the most appropriate OHL outcome measurement instruments, the Consensus-Based Standards for the Selection of Health Measurement Instruments (COSMIN) (30) study design checklist was used. The authors of the English version of the OHLI (15) gave their agreement for the translation into French.

### 2.2 Evaluation of oral health knowledge

An oral health knowledge test based on that of Sabbahi et al. (15) was first used to assess the participants’ level of general dental knowledge. The Oral Health (OH) knowledge test items represent a wide range of dental terms related to anatomical structures and physiological processes, dental materials, devices, treatments and preventive practices. This OH knowledge questionnaire consists of seven images. On each image, the participant had to choose a word and associate it with one of the elements indicated on the image such as peri-oral and intra-oral structures, oral diseases and conditions, dental fillings, dental prosthesis and different oral hygiene tools. The participant had to recognize 17 items. To calculate the final score, each item was scored one point, if answered correctly, or zero points if not (unanswered items were also scored zero points). The sum of these points was then multiplied by 5.88 (100/17) to obtain a final score out of 100. The OH knowledge score was then classified into three levels of knowledge: inadequate (0–59), marginal (60–74) and adequate (75–100).

### 2.3 Elaboration and pre-test of the OHLI-F score

#### 2.3.1 English version of the OHLI

The English version of the OHLI (15) consists of two parts: a first part which assesses the ability to read and understand information about oral diseases (reading comprehension), and a second part that assesses the ability to understand instructions which require basic mathematical operations (numeracy).

The first section consists of two parts, one on dental caries and the other on periodontal disease. The part on dental caries is composed of 13 sentences to complete, with 264 words and 18 words missing in the sentences. The part on periodontal disease is composed of 14 sentences with 228 words and 20 words missing. For each of these 38 missing words, there are 4 proposals but only one is correct. Each correct answer scores one point while an incorrect answer or no answer receives zero points. This section is self-administered and assesses reading comprehension.

The second section consists of a series of printed questions on five prescriptions for drugs frequently prescribed by dentists, a dental appointment card and a post-extraction instructions sheet. This section comprises 19 questions and assesses numeracy. Each correct answer scores one point, while an incorrect answer or no answer receives zero points.

The final score for each section is the sum of all the points for that section. To obtain the final score out of 50 for each section, the total score for the reading comprehension section is multiplied by 1.316 (50/38) and the total score for the numeracy section is multiplied by 2.362 (50/19). The total OHLI score is the sum of these 2 weighted scores together. The total OHLI score varies between 0 and 100. The higher is the score, the higher is the functional competence in oral health. The OHLI score is used to classify three levels of oral health competence: inadequate (0–59), marginal (60–74) and adequate (75–100).

#### 2.3.2 Translation, cross-cultural adaptation of the English version of OHLI

First, the English version of OHLI was translated into French by two bilingual scientists (CC and FC) independently. Then, they compared their two French versions, focusing on the cultural adaptation and discussed the points of divergence in order to reach a consensus and provided a pilot version of the OHLI-F. Secondly, blind back-translation of this pilot version was performed by two bilingual native English speakers with different backgrounds (a public health and education researcher (ADB) and a non-academic professional (PB)). In order to avoid bias, the back-translators were not informed about the concepts covered in the questionnaire and had no access to the original English version of the OHLI. The back-translators compared their two English versions, discussed the points of divergence until they agreed on a consensual version. Thirdly, the two bilingual scientists (CC and FC) compared the back-translated versions with the original English version. In the case of a discrepancy between the 2 English back-translations and the original English version, they (CC and FC) referred to the French versions of the OHLI-F to identify its source. It was then discussed until an agreement was reached. Fourthly, an expert committee composed of 9 members (oral health professionals (DB and NS)), forward translators (CC and FC), backward translators (ADB and PB), health researchers (EV, VEL, BD) reviewed this version and compared it to the original English version of OHLI to determine if they were semantically, idiomatically, experientially, and conceptually equivalent. All discrepancies were resolved through consensus and a pre-final version of OHLI-F was produced.

#### 2.3.3 Pre-test and final version of the OHLI-F

The pre-final version of the OHLI-F questionnaire was tested with a small sample of 6 adults volunteers from different ages and education levels. The 6 volunteers independently completed the questionnaire independently and then gave their feedback. This allowed to identify questions/concepts that were difficult to understand. Then, the expert committee considered their remarks and validated the final version of the OHLI-F.

### 2.4 Psychometric evaluation

Two psychometric assessments were planned: the comparison of OHLI-F scores according to education level and frequency of dental visits, and the test–retest reliability of the OHLI-F.

#### 2.4.1 Study participants

The inclusion criteria for participants were the same in both psychometric evaluations: French citizens, older than 18 years, who could read, write and understand the French language. The common exclusion criteria were people with intellectual disability, visual or auditory acuity problems.

#### 2.4.2 Sample size

Two different sample sizes were calculated.

First, for the comparison of mean OHLI-F scores with education levels or frequency of dental visits, the two-means formula was applied, with alpha = 0.05, power = 80%, standard deviation = 18 (15), expected difference = 10, and expected dropout rate = 20%, resulting in *n* = 51 per group. Since level of education and frequency of dental visit were classified into 3 groups, the total sample size required was 191 participants.

Secondly, to assess test–retest reliability, the sample size needed for the calculation of the intraclass correlation coefficient (ICC) was calculated using the formula of Walter et al. (31). For two replicates, with alpha = 0.05, power = 80%, lower limit of acceptable ICC = 0.6, expected ICC = 0.8, and expected dropout rate = 20%, the total expected sample size was 49 participants.

#### 2.4.3 Study process

The researcher team of the study recruited participants using personal contacts, social networks, and e-mail, so as to have the minimum number of participants for each level of education. The researchers proposed the study to 675 people and asked them if they could read, speak and understand French (well, poorly or not at all). Only those who said they could read, speak, and understand French well and who agreed to participate, were included.

The participants were asked to complete a questionnaire about their age, sex, education level and frequency of dental visits, then they completed OH knowledge and OHLI-F questionnaires.

#### 2.4.4 Statistical evaluation

The level of education was classified as level I or low (< baccalaureate), level II or moderate (from baccalaureate to baccalaureate +2  years) and level III or high (> baccalaureate +2 years). The frequency of dental visit was categorized into three categories: every year, every 2 to 3 years, and never or only in case of pain.

Data were analyzed using R (version 3.6.0, The R Foundation for Statistical Computing Platform). Numerical and categorical socio-demographic variables were calculated and expressed as *n* (percent) and mean standard deviation (SD), respectively. Total scores of the OH knowledge and the OHLI-F were calculated and expressed as mean (SD) and 95% confidence intervals (CI).

Comparison of OHLI-F scores with education level, and with last dental visit was performed using the Brown-Forsythe modified F-test and the comparison of means was carried out with the Bonferroni adapted test. The correlation was considered low or null for scores between 0.00 and 0.25, low for scores between 0.26 and 0.49, moderate for scores between 0.50 and 0.69, high for scores between 0.70 and 0.89 and very high for scores between 0.90 and 1.00 (32). The reliability of the OHLI-F was assessed by examining its internal consistency and test–retest reliability by Cronbach’s alpha and ICC (two-way mixed, absolute agreement, single measure respectively). For Cronbach’s alpha values >0.7 corresponded to good reliability (33, 34). For the ICC, agreement was considered poor for scores below 0.4, moderate for scores between 0.40 and 0.59, good for scores between 0.60 and 0.74 and excellent for scores above 0.74 (35).

## 3 Results

### 3.1 Socio-demographic characteristics of the participants

**Figure 1:**
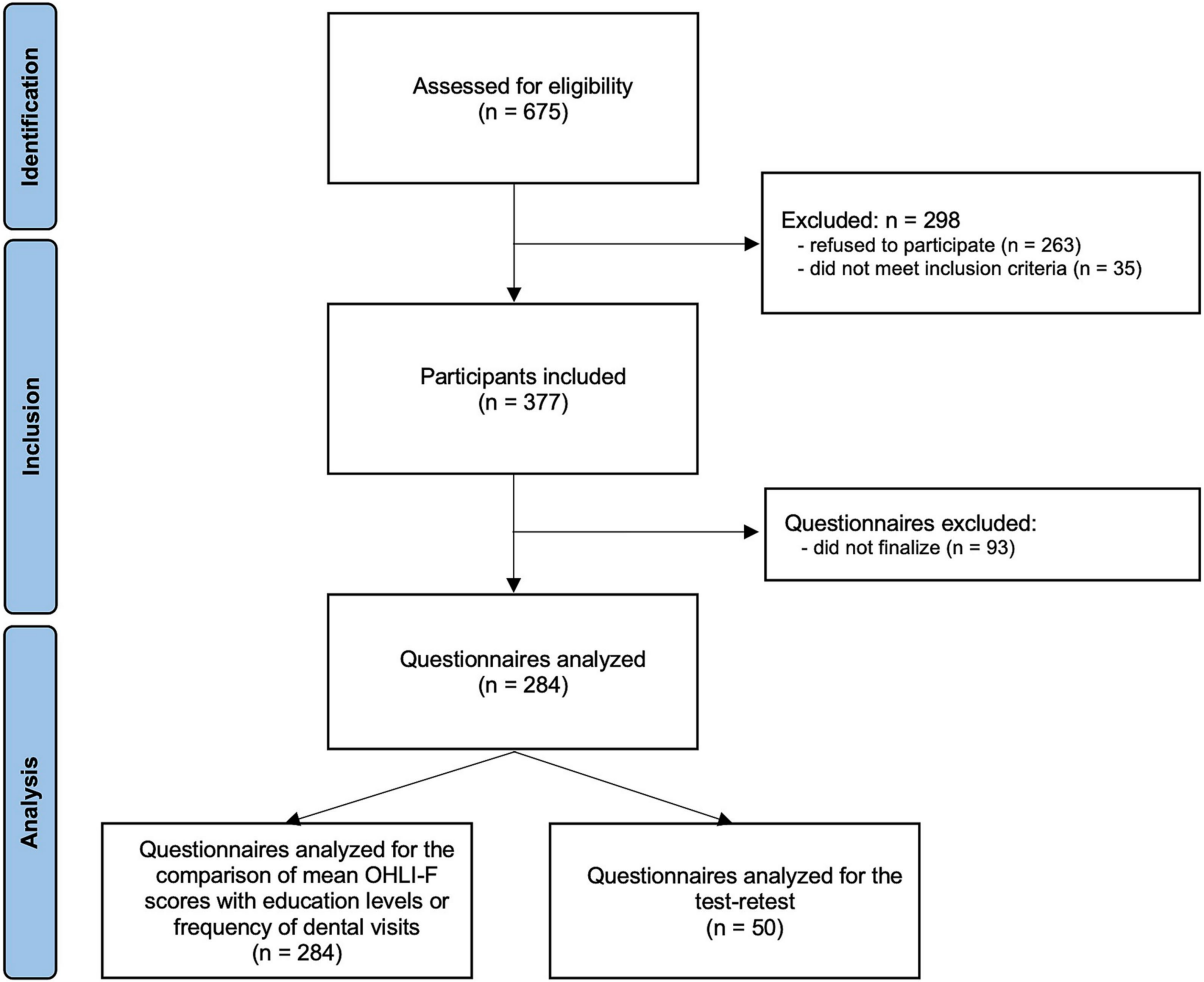
Flowchart.

The flowchart of the study is presented in Figure 1. Among the 675 persons assessed for elligibility, 298 were excluded (263 refused to participate and 35 did not meet inclusion criteria). Thus, 377 were included and 284 completed the OH knowledge and OHLI-F questionnaires. Fifty of these participants completed the OHLI-F after 1 week for the test–retest.

Table 1 describes the sociodemographic characteristics of the participants. Participants had a mean age of 45.64 years (SD = 14.70). Among the participants 59.86% were women (*n* = 170). Participants’ level of education was mainly level III (48.24%, *n* = 137). Most participants stated they visited a dentist every year (58.10%).

**Table 1:** Sociodemographic characteristics of the participants (*n* = 284).

**Variables**	***n* (%)**
Age [years; mean (SD)]	45.64 [14.70]
Age (category)	
<30	52 (18.31)
30–39	47 (16.55)
40–49	63 (22.18)
50–59	68 (23.94)
≥60	54 (19.01)
Gender	
Male	114 (40.14)
Female	170 (59.86)
Education	
Level I	75 (26.41)
Level II	72 (25.35)
Level III	137 (48.24)
Frequency of dental visit	
Every year	165 (58.10)
Every 2–3 years	73 (25.70)
Never or only in case of pain	46 (16.20)

### 3.2 Results of OHLI-F and oral health knowledge scores of all participants

Descriptive statistics for the OHLI-F and OH knowledge questionnaire are shown in Table 2. OHLI-F ranged from 43.42 to 98.68 with a mean value of 75.54. OHLI-F adequate level was obtained for 65.85% of participants (*n* = 187). For the reading comprehension section, the participants obtained a mean score of 41.38 (SD 6.36) while, for the numeracy section, they obtained a mean score of 34.16 (SD 8.46). Oral health knowledge was adequate for 67.61% of participants and the mean score was 79.77 (SD 15.32).

**Table 2:** Descriptive statistics for the OHLI-F scores (*n* = 284).

	**OHLI-F**	**Oral health knowledge**
Total		
Mean (SD)	75.54 (13.59)	79.77 (15.32)
95% CI	73.96–77.12	77.98–81.55
Min - Max	43.42–98.68	35.29–100
Level, *n* (%)		
Inadequate (0–59)	58 (20.42)	39 (13.73)
Marginal (60–74)	50 (17.61)	53 (18.66)
Adequate (75–100)	187 (65.85)	192 (67.61)
Reading comprehension section		NA
Mean (SD)	41.38 (6.36)	NA
95% CI	40.64–42.12	NA
Min - Max	25.00–50.00	NA
Numeracy section		NA
Mean (SD)	34.16 (8.46)	NA
95% CI	33.18–35.15	NA
Min - Max	13.16–50	NA

### 3.3 OHLI-F psychometric properties

#### 3.3.1 Scores according to the level of education and the frequency of dental visit

Table 3 presents the OHLI-F scores according to the level of education and the frequency of dental visit. Based on education level and time since the last dental visit, OHLI-F scores were significantly different between participants. Participants with the lower education level (level I) had significantly lower OHLI-F scores than participants with other education levels (level II and level III). In addition, participants who had visited a dentist in the previous year had significantly higher OHLI-F scores than participants who had never visited a dentist or only in case of pain.

**Table 3:** Analysis of total OHLI-F scores by education level and frequency of dental visit (*n* = 284).

**Variable**	***n***	**Mean (SD)**	***F*-Statistic**^**1**^ **(df1, df2)**	***p*-value**
Education			428,00 (2,17)	1.01. 10–7
Level I	75	56.40 (8.66)		
Level II	72	77.16 (5.50)		
Level III	137	85.17 (5.46)		
Frequency of dental visit			6.19 (3.11)	0.00063
Every year	165	75.74 (16.23)		
Every 2–3 years	73	78.46 (12.35)		
Never or only in case of pain	43	74.57 (12.54)		

^1^Brown-Forsythe modified *F*-test was used due to violation of equal variances assumption. OHLI-F, French version of oral health literacy instrument; SD, standard deviation.

Table 4 compares the OHLI-F total mean scores according to the level of education and frequency of dental visit. Comparisons of OHLI-F total mean scores by pairs of level of education and by pairs of frequency of dental visit, were significantly different.

**Table 4:** Comparison of OHLI-F total mean scores between groups of education level and frequency of dental visit (*n* = 284).

Variable	*p*-value^1^
Education	
Level I - Level II	3.019751. 10–67
Level I - Level III	3.019751. 10–67
Level II - Level III	3.019751. 10–67
Frequency of dental visit	
3–6 months - every year	0.003807968
3–6 months - 2–3 years	0.003807968
3–6 months - never or only in case of pain	0.003807968
Every year - 2–3 years	0.003807968
Every year - never or only in case of pain	0.003807968
2–3 years - never or only in case of pain	0.003807968

^1^Bonferroni correction (alpha = 0.05).

#### 3.3.2 Internal consistency and test–retest reliability of OHLI-F

The results corresponding to the OHLI-F test–retest are presented in Table 5. The internal consistency reliability and test–retest reliability of the OHLI-F were high with a Cronbach’s alpha of 0.881–0.914 and very high with an ICC of 0.985–0.996.

**Table 5:** Internal consistency (by Cronbach’s alpha, *n* = 284) and test–retest reliability (intraclass correlation, *n* = 50) of OHLI-F.

OHLI-F	Cronbach’s alpha (95% CI)	ICC (95% CI)^1^
Reading comprehension section	0.881 (0,784-0,833)	0.996 (0,993-0.998)
Numeracy section	0.914 (0.901–0.926)	0.985 (0,973-0.991)
Total	0.897 (0.886–0.906)	0.995 (0,991-0.997)

^1^Two-way mixed model, absolute agreement. CI, confidence interval; ICC, intraclass correlation coefficient; OHLI-F, French version of oral health literacy instrument.

### 3.4 Oral health knowledge, OHLI-F, and behavioral factors

**Figure 2:**
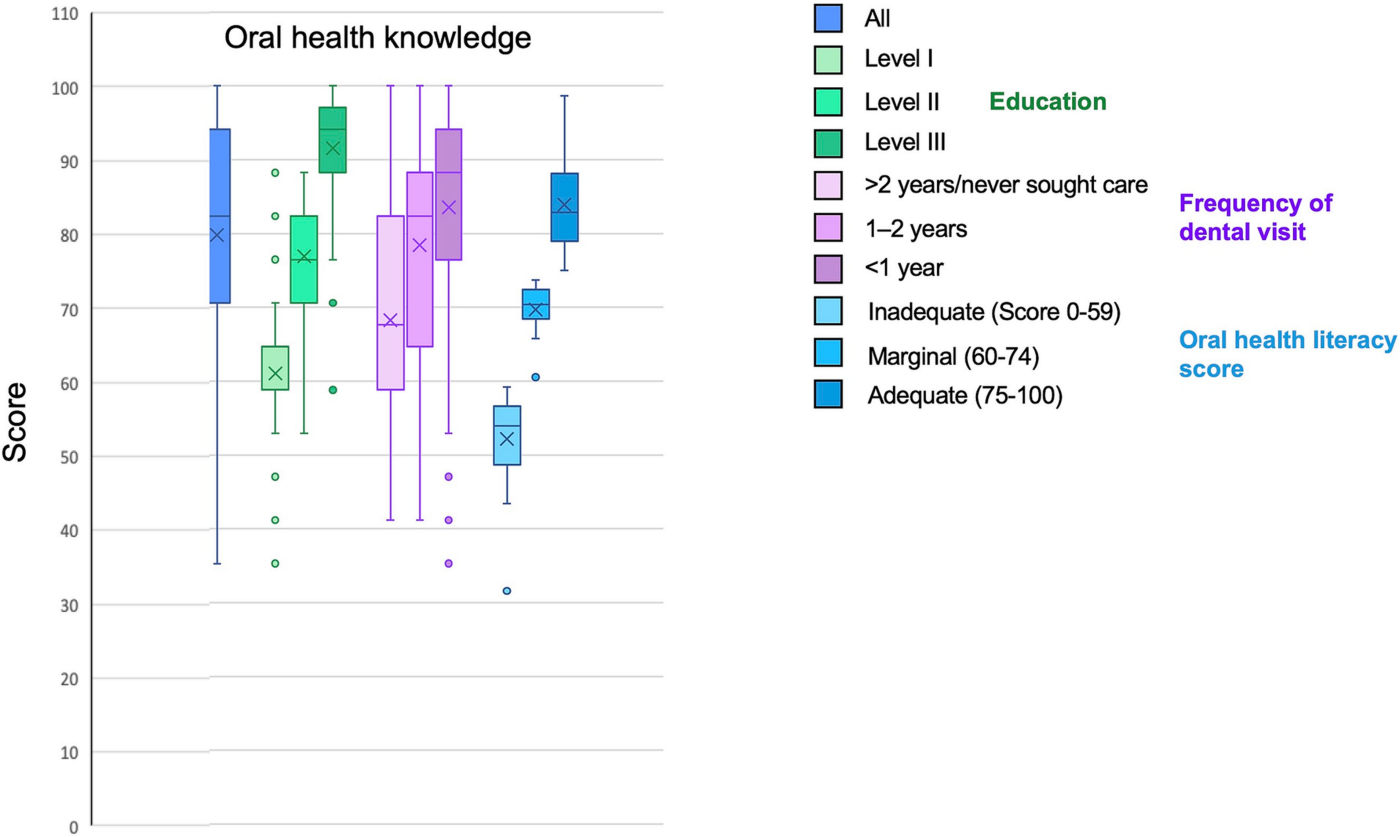
Oral health knowledge depending on behavioral habits and oral health literacy score.

The results obtained through the OH knowledge questionnaire are shown in Figure 2. The scores increased with the education level, the frequency of dental visit and the OHLI-F score.

**Figure 3:**
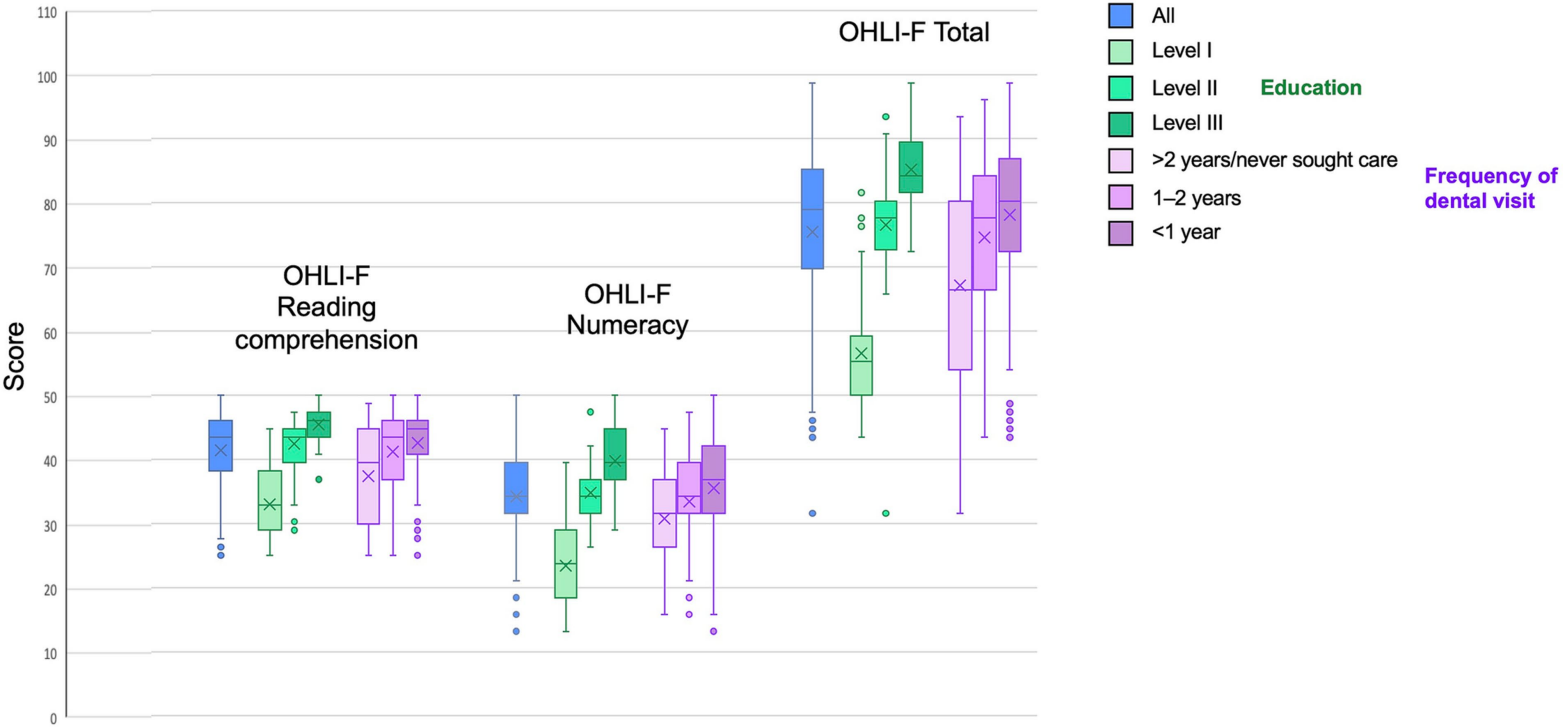
OHLI-F reading comprehension, OHLI-F numeracy skills and OHLI-F total according to behavioral habits.

Oral health knowledge, OHLI-F reading comprehension, OHLI-F numeracy skills and OHLI-F total according to behavioral are represented in Figure 3. The scores increase with the education level and frequency of dental visit.

Table 6 demonstrates that structuring the sample population by level of education explained 66.9% of the variations in the reading comprehension score, 65% in the numeracy score and 78.2% in the OHLI-F score (Table 5). Structuring the population according to the frequency of visits to the dentist explained the 8.5% variation in the reading comprehension score, the 4.9% variation in the numeracy score and the 7.9% variation in the OHLI-F scores. In addition, oral health knowledge was highly correlated to reading comprehension (*r*: 0.789; *ρ*: 0.737), numeracy skills (*r*: 0.726; *ρ*: 0.711) and total OHLI-F scores (*r*: 0.822; *ρ*: 0.786).

**Table 6:** Correlation between total OHLI-F scores (total and sub-section) and associated factors (*n* = 284).

Section	Education level	Frequency of dental visit	Oral health knowledge
Reading comprehension section	0.669	0.085	*r*: 0.789 *ρ*: 0.737
Numeracy section	0.650	0.049	*r*: 0.726 *ρ*: 0.711
Total OHLI-F	0.782	0.079	*r*: 0.822 *ρ*: 0.786

*r*: Pearson correlation; *ρ*: Spearman correlation. *p < 0.05.

## 4 Discussion

Oral health is a global burden and consequently a public health challenge (4). Particularly, in France in 2019, the prevalence of untreated caries on permanent teeth in people aged over 5 was 36.8%, the prevalence of severe periodontal disease in people aged over 15 was 16.2% and the prevalence of edentulism in people aged over 20 was 12.6% (5). To fight this burden, it is essential to improve the level of oral health knowledge. Thus, the availability of a robust tool to assess OHL levels in French should help to reduce oral disease rates and improve overall health, as oral diseases and major non-communicable diseases are interconnected and share the same risk factors (36–38).

OHL is defined as the “degree to which individuals have the capacity to obtain, process and understand basic oral health information and services needed to make appropriate health decisions” (39). This definition emphasizes the importance of comprehension in maintaining oral health and, therefore, that OHL must be patient-centered, as patients are the best managers of their own health (40). A low level of OHL reduces the capacity to understand dentists’ instructions, which affects the maintenance of oral health (41). Data demonstrated that increasing OHL prevents and reduces the prevalence of oral diseases (11, 42). Determining the level of OHL is important to identify people with low OHL and decrease OH inequalities by developing appropriate public health programs, through the creation of adequate educational materials and targeted actions (43). Thus, it could be interesting to offer online material that patients find easy to understand and follow, and in particular audiovisual aids, which are considered effective in improving patients’ knowledge (44). The validated Patient Education Materials Assessment Tool (PEMAT) can be used to assess the understandability and actionability of printable and audiovisual materials on diverse topics and, consequently, it can help health professionals to select patient education materials that reduce health literacy demands (45). In addition, as more and more patients use the Internet to access standardized health information, it is important to control the quality of the information. The online patient education system is thus interesting as it enables clinicians to provide evidence-based, personalized health information. Patients have access to texts and videos adapted to their linguistic, visual and auditory preferences (46).

In 2022, French speakers accounted for 321 million people in the world (18) but, to our knowledge, no OHL instruments are available in French (25). The lack of an instrument to measure health literacy is a cause for concern, as it is a major obstacle to identifying people with low levels of oral health literacy, and it contributes to inequalities. This study addresses this issue. The translation, cultural adaptation and validation of the OHLI in French will enable us to assess oral literacy levels in France and other French-speaking countries. This therefore aligns with WHO recommendations, which explain that “Efforts to raise health literacy will be crucial in whether the social, economic and environmental ambitions of the 2030 Agenda for Sustainable Development are fully realized” (47).

The choice of the OHL instrument to be translated, cross-culturally adapted and validated, was based on an OHL instrument available in English-speaking countries. Several OHL instruments exist but the most widely used are based on HL instruments, either the Rapid Estimate of Adult Literacy in Medicine (REALM) (48) or the Test of Functional Health Literacy in Adults (ToFHLA) (49). The main difference between these HL instruments lies in the fact that REALM is a word recognition test only, which assesses participants’ ability to read from a list of medical terms and estimates reading ability in relation to grade level; whereas ToFHLA assesses participants’ literacy and numeracy skills. According to the systematic review by Praveen et al. (25), the most commonly used OHL instruments for English speakers are: the REALD-30 (14), the REALD-99 (50), the ToFHLiD (16) and the OHLI (15). Among these instruments, the OHLI was selected for the French translation, cross-cultural adaptation and validation for three main reasons. First, contrary to REALD, the OHLI assesses both literacy and numeracy skills. Secondly, although TOFHLiD demonstrated a good convergent validity, it had a moderate ability to discriminate between oral and global HL (16). Finally, the psychometric properties of the OHLI were rated as adequate (15, 28).

The quality of the translation, cultural adaptation and validation from English to French was ensured by following reference methodologies. First, the Guidelines for the Process of Cross-Cultural Adaptations of Self-Report Measures (21) were followed. As recommended, an initial translation, synthesis of translation, back-translation, reviews by an expert committee, and a pre-test version of the instrument were performed. Secondly, for the evaluation of the psychometric properties of the OHLI-F, the COSMIN checklist was used (30, 51, 52). To maintain the original format of the OHLI, only minimal modifications were introduced in the reading comprehension section to adapt to the French context. Modifications were introduced to answer items by substituting them with conceptually similar words or terms, words that are appropriate culturally and adapted to the French health system (e.g., names of French diplomas, the word which corresponds to the profession of dental hygienist which does not yet exist in France has been removed). In the numeracy section, regarding the five prescription labels, the dentist appointment label and the questions relating to these labels from the original OHLI were retained, as the drugs prescribed were similar to those on prescriptions possibly made by dentists in France. With regard to amoxicillin quantities, although they were not exactly those recommended in France (53), the authors retained the initial OHLI frames, considering that the essential questions were the calculation of the intake times and the time between two intakes.

In our study, a statistical association was observed between the OHLI-F total score and OH knowledge as in the English version (15), the Spanish version and the Russian version (27). Thus, OH knowledge could be predictive of OHL level as in Baker’s model (54). In this model, conceptual health knowledge is seen as a necessary foundation for an individual’s health literacy. However, our results do not rule out that health knowledge (vocabulary and conceptual knowledge) could constitute a domain of health literacy, as suggested by the Institute of Medicine’s Expert Panel on Health Literacy (55).

In terms of predictive validity, the OHLI-F is validated because it predicts a correlated measure. Thus, when the OHLI-F was tested, groups of participants were predefined according to levels of education. This enabled the validation of the OHLI-F predefined group analysis. The OHLI-F scores (numeracy, reading comprehension and total) increased with the level of education. In addition, participants who went to the dentist “only when in pain” had the lowest mean score, while those who went every 2–3 years had a higher score, and those who went to the dentist within the last 12 months had the highest score. These results are consistent with those obtained for the English OHLI (15), the Russian OHLI (27) and the Malaysian OHLI (28), which showed a significant difference in OHLI scores according to education level and frequency of dental visits or date of last dental visit. Higher OHLI scores (reading comprehension, numeracy skills and total) were observed in participants with a higher level of education and whose last dental visit was less than a year ago. Comparison with the Spanish OHLI was not possible, as the authors did not analyze frequency of dental visits and level of education (26).

In terms of internal consistency, OHLI-F has very high results for the reading comprehension, numeracy skills and total score. Indeed, Cronbach’s alpha was excellent with values higher than 0.811. Results for the reading comprehension section, numeracy skills and the total score were in line with those of the English (15), Russian (27), Spanish (26) and Malaysian (28) versions. In addition, Cronbach’s alpha value for the numeracy section was higher than for the reading comprehension section, as observed for the Russian version (27), but in contrast to the results observed for the OHLI in English (15), in Spanish (26). This result indicates that, statistically, the numeracy section measures and reading comprehension section were well-defined concept. To conclude, the consistency of the OHLI-F was high.

The temporal validity of OHLI-F was high. Indeed, the results of the test–retest demonstrated excellent agreement for reading comprehension, numeracy skills and total scores. These results are in agreement with those observed for the English (15), Russian (27), Spanish (26) and Malaysian (28) OHLIs.

However, this study had some limitations. First, no other validated Oral Health Literacy instrument in French was available and it was therefore impossible to compare the results and analyze the convergent validity of OHL instruments. Secondly, the sample was not strictly representative of the French population because it was obtained by convenience. Thirdly, given that this version was produced by native French speakers living in France, specific idiomatic and linguistic adaptation may be necessary to ensure perfect comprehension by French speakers living outside France.

## 5 Conclusion

This study provides a robust instrument for assessing OHL level in French (OHLI-F), in terms of reliability and validity. Indeed, oral diseases are a major public health issue in France and other French-speaking countries, notably due to inequalities. Thus, the OHLI-F could facilitate the identification of people most at risk, and to promote oral health through the implementation of appropriate health promotion and education programs.

## Data availability statement

The original contributions presented in the study are included in the article/supplementary material, further inquiries can be directed to the corresponding author.

## Author contributions

CC: Investigation, Methodology, Writing – original draft, Writing – review & editing. V-EL: Investigation, Methodology, Writing – original draft, Writing – review & editing. EV: Investigation, Writing – original draft, Writing – review & editing. BS: Conceptualization, Investigation, Methodology, Writing – original draft, Writing – review & editing. AD-B: Writing – original draft, Writing – review & editing. DB: Writing – original draft, Writing – review & editing. ML: Data curation, Formal analysis, Writing – review & editing. FV: Writing – original draft, Writing – review & editing. CD: Writing – original draft, Writing – review & editing. FC: Conceptualization, Investigation, Methodology, Supervision, Writing – original draft, Writing – review & editing.
